# Increased expression of cancer-associated fibroblast markers at the invasive front and its association with tumor-stroma ratio in colorectal cancer

**DOI:** 10.1186/s12885-019-5462-2

**Published:** 2019-03-29

**Authors:** Tessa P. Sandberg, Maaike P. M. E. Stuart, Jan Oosting, Rob A. E. M. Tollenaar, Cornelis F. M. Sier, Wilma E. Mesker

**Affiliations:** 1 Department of Surgery, Leiden University Medical Center, Albinusdreef 2, 2333ZA Leiden, the Netherlands; 2 Department of Pathology, Leiden University Medical Center, Albinusdreef 2, 2333ZA Leiden, the Netherlands

**Keywords:** Cancer-associated fibroblasts, Tumor microenvironment, Tumor-stroma ratio, FAP, Colorectal cancer

## Abstract

**Background:**

The tumor microenvironment has a critical role in regulating cancer cell behavior. Tumors with high stromal content are associated with poor patient outcome. The tumor-stroma ratio (TSR) identifies colorectal cancers (CRC) with poor patient prognosis based on hematoxylin & eosin stained sections. The desmoplastic reaction consists to a great extent of cancer-associated fibroblasts (CAFs) of which different subtypes are known. The aim of this study is to investigate and quantify CAFs present in the tumor stroma of CRC stratified by the TSR to possibly add prognostic significance to the TSR.

**Methods:**

The expression of established CAF markers was compared between stroma-low and stroma-high tumors using transcriptomic data of 71 stage I – III CRC. Based on literature, fibroblast and stromal markers were selected to perform multiplex immunofluorescent staining on formalin fixed, paraffin-embedded tumor sections of patients diagnosed with stage III colon cancer. Antibodies against the following markers were used: αSMA, PDGFR -β, FAP, FSP1 and the stromal markers CD45 and CD31 as reference. The markers were subsequently quantified in the stroma using the Vectra imaging microscope.

**Results:**

The transcriptomic data showed that all CAF markers except one were higher expressed in stroma-high compared to stroma-low tumors. Histologically, stroma-high tumors showed a decreased number of FSP1^+^/CD45^+^ cells and a trend of an increased expression of FAP compared to stroma-low tumors. FAP was higher expressed at the invasive part compared to the tumor center in both stroma-high and stroma-low tumors.

**Conclusions:**

The increased expression of FAP at the invasive part and in stroma-high tumors might contribute to the invasive behavior of cancer cells. Future functional experiments should investigate the contribution of FAP to cancer cell invasion. Combining the quantity of the stroma as defined by the TSR with the activity level of CAFs using the expression of FAP may result in an expanded stroma-based tool for patient stratification.

**Electronic supplementary material:**

The online version of this article (10.1186/s12885-019-5462-2) contains supplementary material, which is available to authorized users.

## Background

The tumor microenvironment or tumor stroma has a critical role in regulating cancer cells. It is involved in tumorigenesis by inducing stem cell-like properties and epithelial-to-mesenchymal transition (EMT) in cancer cells [1]. The tumor-stroma ratio (TSR) is a prognostic tool that stratifies tumors into stroma-low and stroma-high based on the quantity of stroma scored in hematoxylin & eosin (H&E) stained sections [2, 3]. Stroma-high tumors were shown to have a poor patient prognosis in colorectal cancer as well as in other solid epithelial tumors [4–7]. Additionally to the quantity of the tumor stroma, the composition of the stroma may be an important determinant of cancer behavior. Several transcriptomic and immunohistochemical studies have shown that desmoplastic stroma is associated with poor patient outcome and can predict response to therapy [8–11]. The desmoplastic reaction consists mainly of activated fibroblasts, also called cancer-associated fibroblasts (CAFs). CAFs are a heterogeneous cell population in terms of origin and biological function and derive mainly from mesenchymal cells that are resident or recruited by the tumor [12]. They are situated close to cancer cells and other components of the stroma like immune cells, blood vessels and components of the extracellular matrix (ECM). Fibroblast is a general term which includes resident quiescent fibroblasts, CAFs, myofibroblasts and pericytes. The identification and nomenclature of fibroblasts present in the tumor remain challenging due to the lack of specific markers for known and still undefined subtypes.

The aim of this study is to investigate and quantify CAFs present in the tumor stroma in colorectal cancer (CRC) stratified by the TSR. The composition of the tumor stroma may add prognostic value to the TSR. In a cohort of 71 CRC patients, the difference in transcriptomic data of currently used fibroblast and CAF markers were first compared between stroma-low and stroma-high tumors. From the fibroblast markers investigated, the most commonly used in literature were stained in colon tumor sections of thirty-two stage III patients using multiplex immunofluorescence microscopy [13, 14]. PDGFRβ, FAP, FSP1 and αSMA were selected as markers for CAF subtypes. During evaluation of the markers, it became clear that FSP1 showed a different staining pattern compared to the other fibroblast markers. Based on the preliminary data, the fibroblast markers were divided into two panels: the first panel included the markers FAP, PDGFRβ, αSMA and CD31. The panel was called ECM-related fibroblast markers as it identified activated fibroblast involved in creating and remodeling the ECM. The second panel consisted of FSP1, CD45 and αSMA and was called immune-related fibroblast markers as it included CD45 staining for immune cells. After quantifying the markers in an automated manner using the Vectra microscope, the staining results were compared between stroma-low and stroma-high tumors on the one hand and between the tumor center versus the most invasive part of the tumor on the other hand.

## Methods

### Patient material

The LUMC cohort consisted of 71 CRC tissues of patients diagnosed with TNM stage I – III who underwent surgery at the Leiden University Medical Center (LUMC) between 1991 and 2005. The patients were not treated with (neo-)adjuvant therapy. This cohort was previously analyzed as part of a larger cohort [15]. Formalin fixed, paraffin-embedded (FFPE) whole sections of the tumors were not available for immunofluorescence staining.

A second cohort was therefore used to perform immunofluorescence staining on FFPE whole sections and consisted of 32 patients diagnosed with stage III colon cancer who underwent surgery at the LUMC. Stage III tumors were selected as these tumors have an increased proportion of stroma-high tumors with more CAFs compared to lower stages tumors. None of the patients received preoperative anticancer therapy. Detailed patient characteristics of both cohorts can be found in Additional file 1: Table S1.

### Transcriptomic analysis

RNA was previously isolated from fresh frozen tissue, hybridized to a customized Agendia oligonucleotide array and normalized as described elsewhere [15].

### Immunofluorescence staining

The antibodies were first optimized using single fluorescence staining (Table 1). Various FAP antibodies (AF3715 R&D, ab53066, ab28244 Abcam) were evaluated (data not shown) due to inconsistency with the literature and the selected antibody was confirmed by immunohistochemistry and western blot (Additional file 1: Figure S3). Immunofluorescence staining was performed on two 5 μm histological tissue sections of each tumor. After deparaffinization and rehydration, the sections underwent antigen retrieval by heating during 10 min at 95 °C in pH high Target Retrieval Solution (Dako). Unspecific protein binding sites were blocked with protein block (Dako) for 15 min. Sections were incubated overnight with the primary antibodies at specific dilutions described in Table 1. The following day, appropriate AlexaFluor secondary antibodies (ThermoFisher Scientific, 1:200) were applied to the sections for one hour. Sections were counterstained with DAPI (2μg/ml, Sigma-Aldrich) and mounted in ProLong Gold Antifade Mountant (ThermoFisher Scientific). The immunohistochemical staining was performed according to a similar protocol. Following deparaffinization and rehydration, sections were incubated with 0.3% hydrogen peroxide solution (Millipore) to block endogenous peroxidase. Then, after incubation with the primary antibody FAP (AF3715 R&D, 1:400), the sections were incubated with a HRP-labelled secondary antibody donkey anti-sheep (Invitrogen, A16041), then they were developed with DAB chromogen (Dako) and finally the sections were counterstained with hematoxylin.

**Table 1:** Characteristics of antibodies

	Artificial color	Clone	Provider	Concentration	Origin	Secondary antibody
Extracellular matrix-related fibroblast markers						
DAPI			Sigma-Aldrich	2μg/ml		
PDGFR-β		3169S	Cell Signaling	1:50	Rabbit	AlexaFluor 488
FAP		AF3715	R&D Systems	1:20	Sheep	AlexaFluor 546
CD31		JC70A	Dako	1:1000	Mouse IgG1	AlexaFluor 594
α-SMA		1A4	Dako	1:100	Mouse IgG2a	AlexaFluor 680
Immune-related fibroblast markers						
DAPI			Sigma-Aldrich	2μg/ml		
CD45		PD7/2 + 2B11	Dako	1:1000	Mouse IgG1	AlexaFluor 514
FSP1		D9F9F	Cell Signaling	1:1000	Rabbit	AlexaFluor 594
α-SMA		1A4	Dako	1:100	Mouse IgG2a	AlexaFluor 680

### Microscopical analysis

#### Spectral library and background correction

For each antibody panel, a single-stained section was prepared for every marker (including DAPI) to build a library containing the emitting spectral peak of each fluorophore in InForm (PerkinElmer, 2.2.1) (Additional file 1: Figure S1 A and B). Unstained colon cancer tissues were used as negative controls to perform background correction.

#### Multispectral imaging and spectral unmixing

Multiplexed stained sections were imaged using the VECTRA 3.0 multispectral sections imaging system (PerkinElmer, 3.0.4). Using PhenoChart software (PerkinElmer, 1.0.4), a maximum of 8 multispectral imaging fields of 334 × 250 μm (40x field) were selected in the tumor center and at the invasive part (Additional file 1: Figure S2B). The selection of the 40x fields was based on the annotations of a pathologist on H&E stained sections. Filter cubes used for multispectral imaging were DAPI (440–680 nm), FITC (520 – 680 nm), Cy3 (570 – 690 nm), Texas Red (580 – 700 nm) and Cy5 (670 – 720 nm). Next, spectral unmixing was performed on the extracted profiles using the spectral library by InForm (PerkinElmer, 2.2.1). Following background correction using two negative control sections, the unmixed images were quantified for pixel count or phenotype (see below).

#### Quantification of pixel count – ECM-related fibroblast markers

Following background correction, the absolute intensity of pixel counts per marker was determined in the spectrally unmixed images. An active learning algorithm was developed to segment the tissue (“Stroma”, “Tumor” and “Empty”) based on 40x fields of tumors originating from different patients (Additional file 1: Figure S2C). The total number of pixels present in the stroma was used to normalize the data.

Regarding the ECM-related fibroblast panel, 29 colon tumors and 392 40x fields were analyzed. The tumor center was analyzed in 27 colon tumors and the invasive part was analyzed in 25 tumors.

#### Quantification of cell count – immune-related fibroblast markers

A second learning algorithm was developed to count the number of DAPI-stained cells with different phenotypes. The algorithm was prepared as follows: background correction was performed, tissue segmentation (“Stroma”, “Tumor” and “Empty”) was performed on the 40x fields and cell segmentation was assessed on the DAPI-stained cells. The algorithm was then trained on 40x fields originating from various patients (Additional file 1: Figure S2C). Cells were phenotyped and categorized into one of the following classes CD45^+^, FSP1^+^, CD45^+^/FSP1^+^ and CD45^−^/FSP1^−^ (Additional file 1: Figure S2D).

In the immune-related fibroblast panel, 27 colon tumors and 398 40x fields were analyzed. 25 tumors were analyzed in the tumor center and 26 at the invasive part. Only cells present in the stroma, assessed based on the tissue segmentation, were included in the analysis. The spindle-like shape of fibroblasts identified with the ECM-related fibroblast markers did not allow automated counting of the cells by nuclei. These markers were therefore quantified by pixel count while, for the immune-related fibroblast markers, the number of cells were counted using DAPI. The pixels or the cell numbers were averaged according to their location (tumor center versus invasive part) in each tumor.

### Cell culture, lysis and western blot

The human colon fibroblast cell line CCD-18Co (ATCC) was cultured at low passage in EMEM medium with 10% fetal calf serum (FCS). The fibrosarcoma cell line HT1080 and the FAP-transfected HT1080 (a kind gift of R. Kontermann and O. Seifert, University of Stuttgart, Germany) were cultured in DMEM medium supplemented with 10% FCS. The cell lines were mycoplasma tested before use. 1.5 × 10^5 cells of CCD-18Co fibroblasts were seeded in 6 well-plates. The following day, the CCD-18Co cells were stimulated with recombinant human TGFβ1 (5 ng/ml, HEK293 derived, Peprotech) in serum free medium for three days and compared with non-stimulated cells. HT1080 and FAP-transfected HT1080 were used as negative and positive controls, respectively. Both fibroblasts and HT1080 cells were lysed with RIPA buffer (150 mM NaCl, 1% Triton X-100, 0.5% SDS, 50 mM Tris) and a western blot was performed.

Proteins were separated using electrophoresis on a denaturing 10% polyacrylamide gel and transferred to a polyvinylidene difluoride (PVDF) membrane using TurboBlot (Biorad). The primary antibodies against FAP (1:10 000 for HT1080; 1:2500 for CCD-18Co, AF3715 R&D) and beta-actin (1:2000, DM1A Cell Signaling) were applied to the membrane overnight at 4 °C. The membrane was incubated for 1 h with HRP-labeled secondary antibodies anti-sheep HAF016 (1:3000, R&D Systems) or anti-mouse (1:2000, Cell Signaling). The membrane was developed using enhanced chemiluminiscence and exposed to hyperfilm (GE Healthcare Life Sciences).

### Tumor-stroma ratio

The primary tumors of both cohorts were scored for TSR on 5 μm H&E stained tissue sections as described previously [2, 5]. The tissue samples selected were those defined as the most invasive part of the primary tumors as used by the pathologist to determine the T-status. To determine the TSR, the region with the highest stroma was selected using an 2.5x or 5x objective. A microscopy field was scored where tumor cells were present at all borders of the image field (north, south, east, west) of the 10x objective. Scoring percentages were given in 10-fold percentage per image field and the field with the lowest percentage determined the final score. The TSR of the LUMC cohort was scored previously [16] and one investigator (T.P.S.) estimated the stromal percentage of the stage III cohort in a blinded manner.

### Statistical analyses and data analyses

Statistical analyses were performed using IBM SPSS Statistics software (version 23) and the figures were made using GraphPad Prism 7 (version 7.02). A normality test was performed in the transcriptomic data and the mean of each gene in the stroma-low and stroma-high groups was compared using multiple independent t-tests followed by multiple testing correction using False Discovery Rate (FDR) (q = 0.05). Regarding the immunofluorescence data, the pixel count (ECM-related fibroblast markers) and the phenotyping (immune-related fibroblast markers) of each 40x field were normalized by the total number of stromal pixels or the total number of stromal cells, respectively. The normalized data was then averaged by location (tumor center or invasive part) and transformed using the following formula log(p/(1-p)). It was not possible to score all tumors at both the tumor center and the invasive part because of poor quality of the tissue or because the tumor center or invasive part was not present on the tissue (Additional file 2: Supplementary data). Independent student’s t-tests were therefore performed to compare the averaged markers between the location as well as between the stroma-low and stroma-high groups. The mean (M) and the standard error (SE) were reported for each t-test. *P* values lower than 0.05 were considered significant.

## Results

### Transcriptomic analysis of cancer-associated fibroblast markers in stroma-low versus stroma-high tumors

**Fig. 1:**
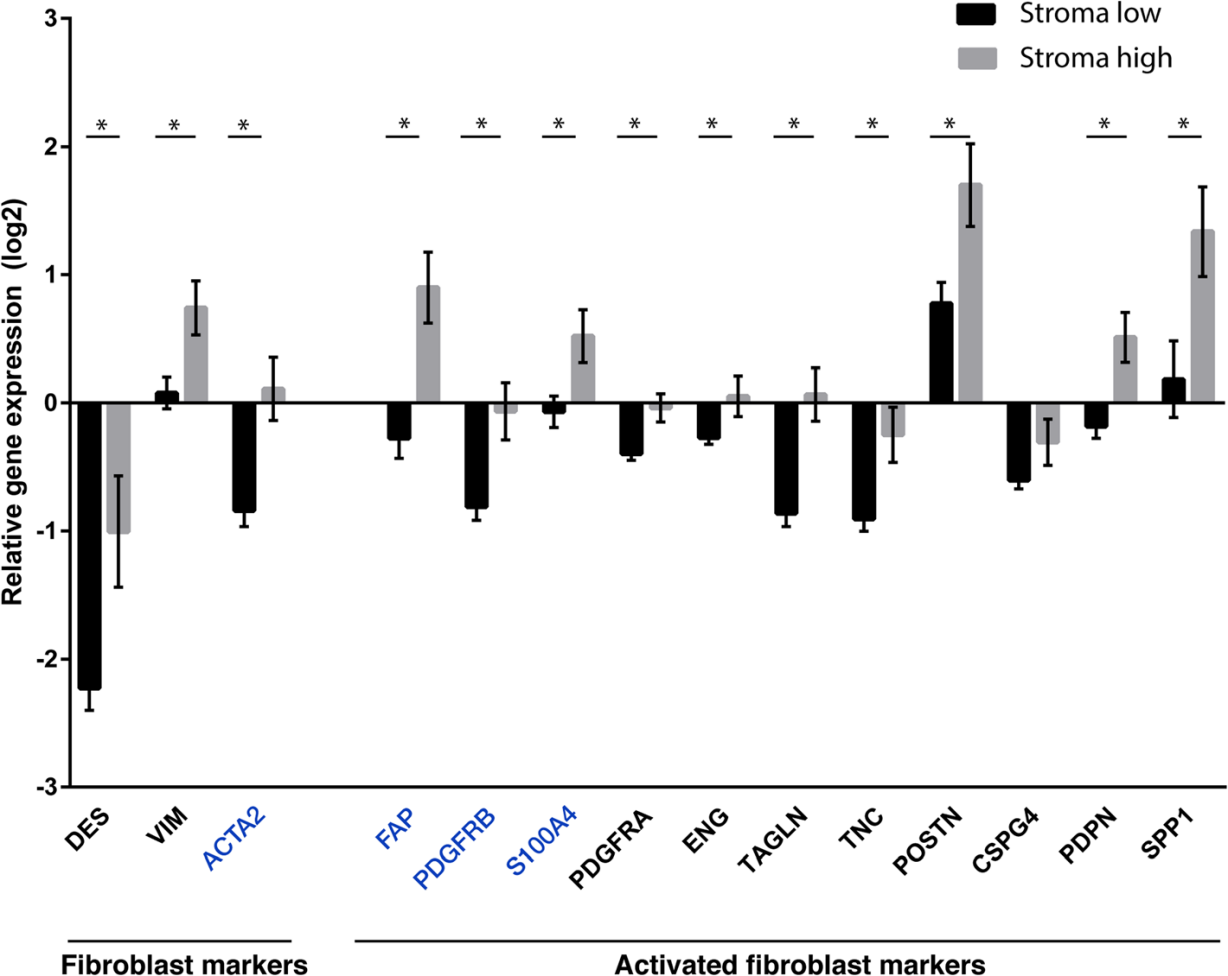
Cancer-associated fibroblast markers stratified by the tumor-stroma ratio in transcriptomic data. Transcriptomic differences of cancer-associated fibroblast markers between stroma-low and stroma-high tumors in the LUMC cohort consisting of 71 colorectal cancer patient. All markers except *CSPG4* were significantly higher expressed in stroma-high compared to stroma-low tumors. The markers in blue were used in immunofluorescence analysis. Multiple independent t-tests followed by False Discovery Rate correction, * q < 0.05, mean ± SE

Fibroblast and CAF markers were selected based on literature and were analyzed in transcriptomic data between stroma-low and stroma-high tumors. Vimentin (*VIM*) was included as a general mesenchymal cell marker and desmin (*DES*) was included as a smooth muscle cell-specific marker. Activated fibroblast markers included αSMA (*ACTA2*), fibroblast activation protein (*FAP*), platelet derived growth factor receptor-α and -β (*PDGFRA*, *PDGFRB*), fibroblast specific protein 1 (*FSP1/S100A4*), endoglin (*ENG*), transgelin (*TAGLN*), tenascin C (*TNC*), periostin (*POSTN*), chondroitin sulphate proteoglycan 4 or neuron-glial antigen 2 (*CSPG4/NG2*), podoplanin (*PDPN*) and osteopontin (*SPP1*). The means of each fibroblast marker showed significant higher expression in stroma-high tumors (*N* = 20) compared to stroma-low tumors (*N* = 51; *P* < 0.05, q < 0.05; Fig. 1). *CSPG4/NG2* was the only marker which was not statistically differently expressed between the two groups. These results confirmed that stroma-high tumors are associated with an increased number of activated fibroblast markers. The abovementioned markers have been described to identify different subpopulations of fibroblasts based on the co-expression with different phenotypic markers. Therefore, immunofluorescence was used to further investigate the co-expression of CAF markers.

### Expression of fibroblast markers αSMA, PDGFRβ, FAP and FSP1 in stage III colon cancer

**Fig. 2:**
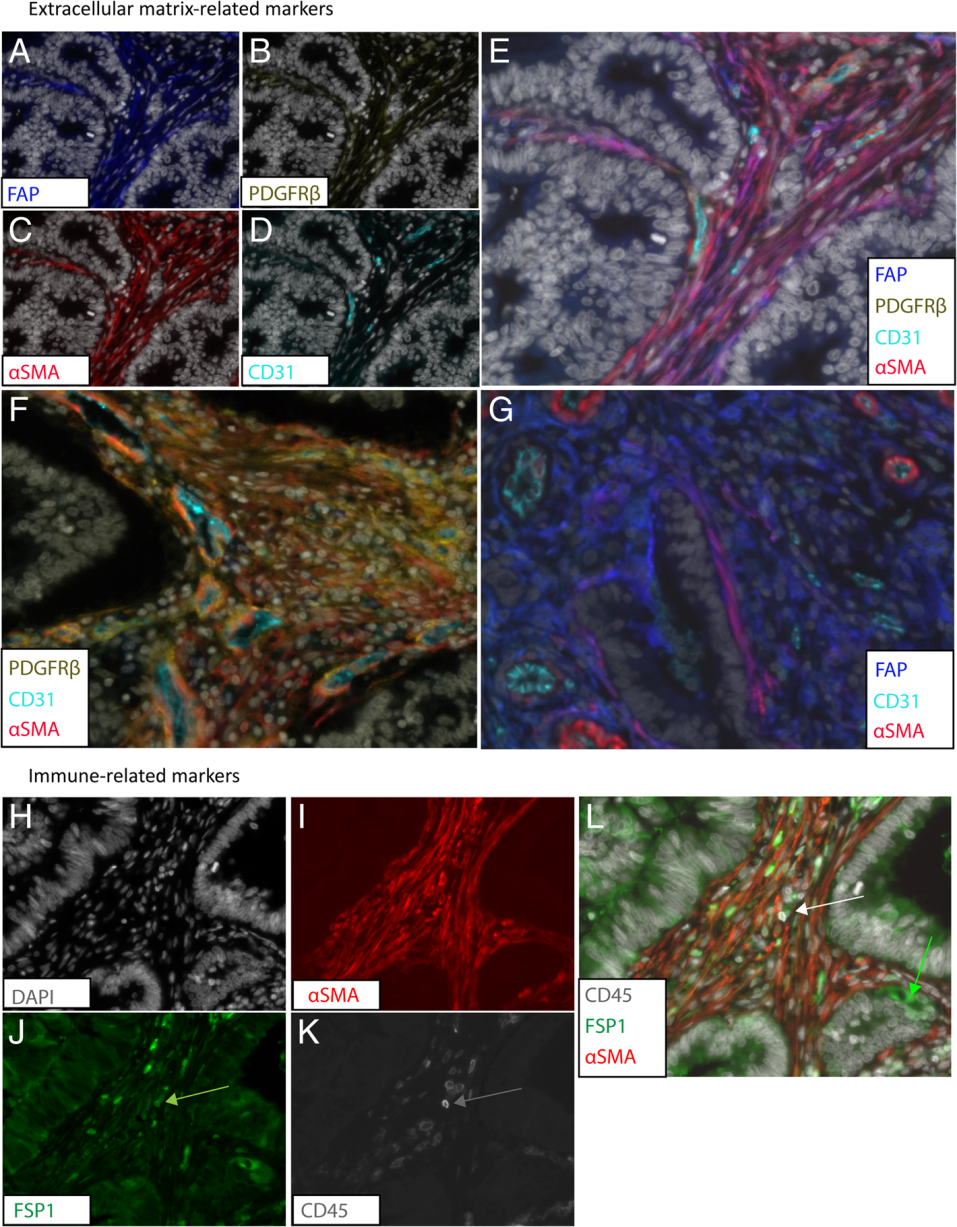
Multiplex analysis of cancer-associated fibroblast markers in a cohort of stage III colon cancer. Representation of enlarged 40x fields of the extracellular matrix-related markers showing the individual markers FAP (**a**), PDGFRβ (**b**), αSMA (**c**) and CD31 (**d**) together with DAPI (grey), and the composite image using the same colors (**e**). Representations showing co-expression (orange) of PDGFRβ and αSMA around CD31(**f**) and co-expression (pink) of FAP and αSMA around tumor cells (**g**). Representation of enlarged 40x fields of the immune-related fibroblast markers showing the individual markers DAPI (**h**), αSMA (**i**), FSP1 (**j**) and CD45 (**k**) together with DAPI and the composite image showing co-expression of CD45 and FSP1 (white arrow, **l**). FSP1 was also expressed in some cancer cells (green arrow, **l**)

Regarding the ECM-related fibroblast markers, FAP, PDGFRβ and αSMA were all exclusively expressed in the stromal compartment (Fig. 2a-c.). All three fibroblast markers co-expressed with each other in some regions and did not co-express with CD31 (Fig. 2d, e). PDGFRβ and αSMA were expressed near CD31^+^ endothelial cells where the two markers co-localized and marked perivascular smooth muscle cells and pericytes (Fig. 2f). FAP was expressed at different levels throughout the stroma and was not expressed near CD31^+^ cells (Fig. 2g). Interestingly, FAP and αSMA co-expressed in a few regions and both markers were expressed individually in different stromal areas (Fig. 2g). This suggests that αSMA^+^ and FAP^+^ combined marked a similar CAF subtype while the presence of the two markers individually characterizes two different cell types. ECM-related markers were expressed in all tumors. However, in the tumor center, we did not detect FAP and CD31 in three tumors and in one tumor, respectively (Additional file 1: Table S2).

Regarding the immune-related fibroblast markers, FSP1 was expressed extracellularly, in the nucleus and in the cytoplasm of stromal cells as well as in the cytoplasm of some epithelial tumor cells (Fig. 2j, l). Stromal FSP1 was not expressed directly adjacent to cancer cells. Immune cells characterized by the CD45 membrane marker were mainly present in the stroma and to a lesser extent between tumor cells (Fig. 2k). There was a high number of FSP1^+^ cells compared to CD45^+^ and FSP1^+^ /CD45^+^ cells. CD45 and FSP1/CD45 were expressed in nearly all tumors while FSP1 was expressed in 76% of the tumor center and 89% of the tumors at the invasive part (Additional file 1: Table S2).

### Comparison of cancer-associated fibroblast markers in stroma-low and stroma-high tumors, and between the tumor center and the invasive part

The fibroblast markers were quantified in an automated manner in the tumor center and at the invasive part according to the workflow depicted in Additional file 1: Figure S2A.

**Fig. 3:**
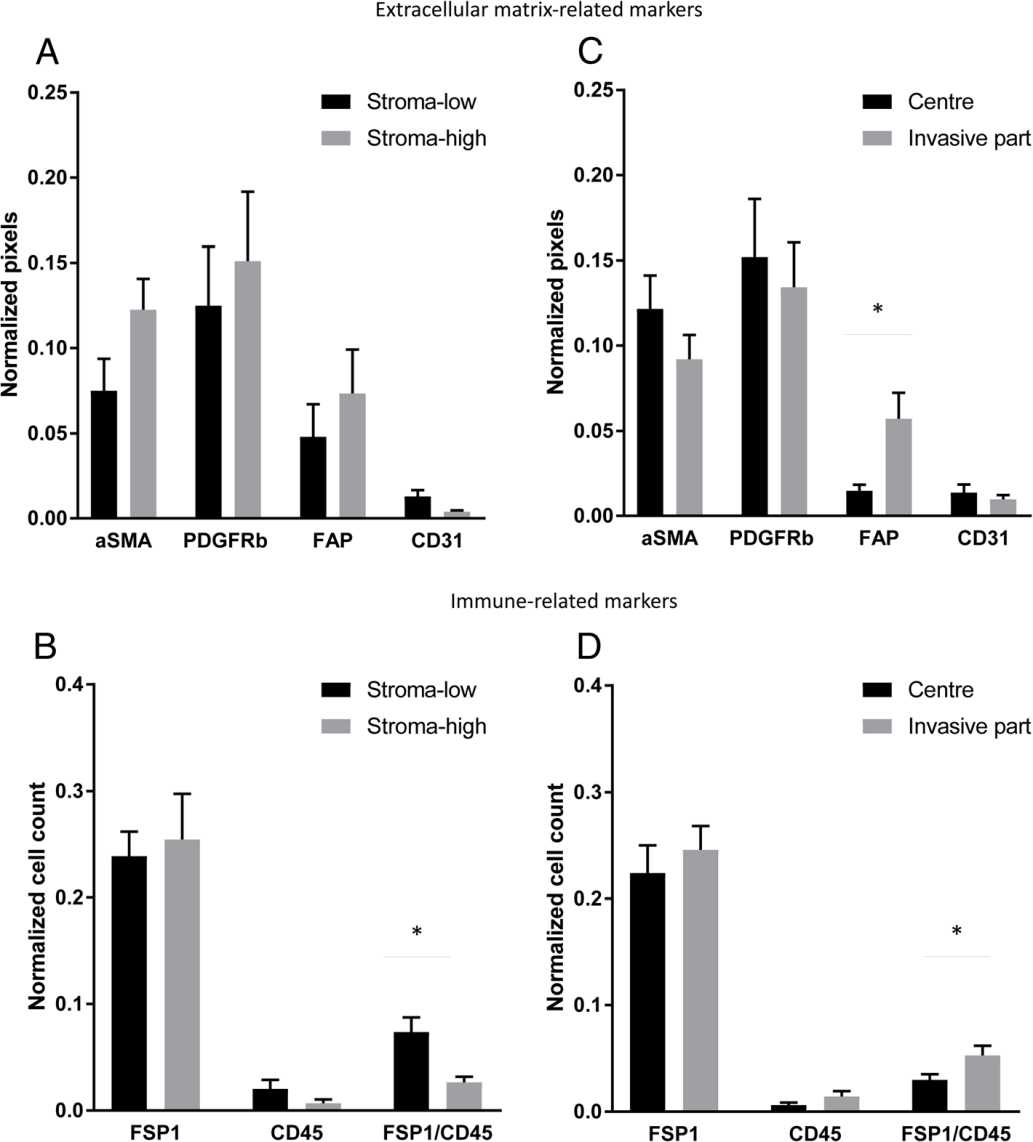
Quantification of extracellular matrix-related and immune-related fibroblast markers in the cohort of stage III colon cancer according to the tumor-stroma ratio and to the location. The markers of the extracellular matrix-related (**a**) and the immune-related (**b**) fibroblast panels expressed at the invasive part of the tumor were averaged according to the tumor-stroma ratio. The extracellular matrix-related fibroblast markers (**c**) and the immune-related fibroblast markers (**d**) were also compared between the tumor center and the invasive part in the whole cohort. Independent t-test on log-transformed data, mean ± SE, * *P* value < 0.05

Because the TSR is assessed at the invasive part of tumors, we compared the CAF markers at the invasive part between the stroma-low (*N* = 16) and the stroma-high groups (*N* = 9). Stroma-high tumors tended to have an increased expression of FAP (M low = 0.048, SE = 0.019; M high = 0.073, SE = 0.026) and a lower expression of CD31 (M low = 0.013, SE = 0.004; M high = 0.004, SE = 0.001) relative to the total amount of stroma, although the difference did not reach statistical significance (Fig. 3a). Additional file 1: Figure S3B. shows the increased FAP expression in CAFs surrounding the tumor cells in a stroma-high tumor. The mean number of CD45^+^ cells and FSP1^+^ cells did not differ while there was an increased number of double positive FSP1^+^/CD45^+^ cells in the stroma-low group (*N* = 15, M = 0.074, SE = 0.014) compared to the stroma-high group relative to the total number of stromal cells (*N* = 11, M = 0.029, SE = 0.006; *P* = 0.003) (Fig. 3b).

As spatial distribution contributes to the activation level of CAFs, the averaged pixel counts of the different markers were compared between the tumor center and the invasive part in the whole cohort. On average, αSMA, PDGFRβ and CD31 were not differently expressed between the tumor center (αSMA M = 0.122, SE = 0.019; PDGFRβ M = 0.152, SE = 0.034; CD31 M = 0.014, SE = 0.005) and the invasive part (αSMA M = 0.092, SE = 0.014; PDGFRβ M = 0.134, SE = 0.026; CD31 M = 0.010, SE = 0.002; *P* > 0.05) (Fig. 3c). No difference was found in the number of cells expressing FSP1 or CD45 between the center (FSP1 M = 0.224, SE = 0.026; CD45 M = 0.006, SE = 0.002) and the invasive part (FSP1 M = 0.246, SE = 0.023; CD45 M = 0.014, SE = 0.005; *P* > 0.05) (Fig. 3d). FAP showed an increased expression at the invasive part (M = 0.057, SE = 0.015) compared to the tumor center (M = 0.015, SE = 0.003; *P* = 0.028) (Fig. 3c). FSP1^+^/CD45^+^ cells were higher expressed at the invasive part (M = 0.053, SE = 0.009) compared to the tumor center (M = 0.030, SE = 0.005; *P* = 0.044) (Fig. 3d).

## Discussion

In the transcriptomic data, almost all CAF markers were higher expressed in stroma-high tumors compared to stroma-low tumors. Of the markers investigated, FAP was the only marker that showed an increased expression in stroma-high tumors using immunofluorescence. FAP expression was also increased at the invasive part compared to the tumor center.

Previous research showed that FAP^+^ CAFs contribute to the invasive behavior of cancer cells [17]. FAP is a cell surface protease expressed predominantly by CAFs. The protein is able to suppress anti-tumor immune response [18]. FAP is involved in ECM remodeling with collagen being a key substrate, herewith facilitating tumor migration [19]. The ability of FAP to affect cancer cell behavior might explain why the present study found an increased expression of FAP at the invasive part and in stroma-high tumors, known to be more aggressive than stroma-low tumors. Furthermore, FAP expression has shown prognostic significance in colon cancer [20]. It would therefore be interesting to investigate the prognostic value of FAP expression using the automated method of this study. Combining the quantity of the stroma as defined by the TSR with the activity level of CAFs using FAP may add prognostic value to the TSR.

Moreover, we found a decreased number of cells co-expressing FSP1/CD45 in stroma-high compared to stroma-low tumors. FSP1 stained round-shaped cells instead of stellate-shaped cells and a part of the FSP1^+^ round cells co-expressed with CD45+ but not with αSMA cells (Table 2). The simplest explanation might be that these FSP1^+^ CD45^+^ cells are a subset of immune cells, most likely macrophages, as has been reported previously in different tissue types [21–23]. The increased presence of this cell subtype in stroma-low tumors might correspond to the better prognosis associated with stroma-low tumors.

**Table 2:** Expression and co-expression in different location and cell types of fibroblast and stromal markers analyzed in this study

Markers	Expression of the marker	Subpopulations
CD31	Blood vessels	Endothelial cells
αSMA	Cancer stroma Blood vessels	CAFs, Smooth muscle cells Myofibroblasts in healthy colon
PDGFRβ	Cancer stroma Blood vessels	CAFs, myofibroblasts, pericytes
FAP	Cancer stroma	CAFs
CD45	Immune cells	Immune cells, hematopoietic stem cells
FSP1	Fibroblasts	Quiescent fibroblasts, cancer cells
PDGFRβ αSMA	Around CD31+ endothelial cells	Pericytes, smooth muscle cells?
	Cancer stroma	CAFs
FAP αSMA	Cancer stroma	CAFs
PDGFRβ αSMA FAP	Cancer stroma	CAFs
CD45 FSP1	Cancer stroma	Macrophages / monocytes / fibroblast precursors or quiescent fibroblasts (?)

The TSR assessed on H&E tissue sections was associated with the transcriptomic expression of fibroblast and CAF markers. However, the transcriptomic results were not exactly in line with the immunofluorescence data, which can be explained by the fact that transcriptomic data was not normalized by the amount of stroma in contrast to the immunofluorescence data. Consequently, an increased expression of a marker in the transcriptomic data can be attributed to either an increased gene expression level or to an increased presence of specific cells (or both). The transcriptomic and immunofluorescence methods should therefore be considered as complementary.

The main limitation of this study was that the algorithms made to quantify the markers still need further optimization. For instance, regarding the ECM-related fibroblast panel, no tumor stroma segmentation step could be implemented in the initial algorithm used for pixel count. An extra algorithm had to be made to normalize the pixel count which generally leads to inaccuracy. The phenotyping in the immune-related fibroblast algorithm was not optimal and underestimated the number of CD45^+^ cells, which might have influenced the results. The reported CD45^+^ cells in the tumor should be considered as relative numbers rather than absolute numbers. The InForm software to develop the algorithms should therefore be further optimized.

The question remains whether the markers used in the present study define subtypes of CAFs performing unique functions. Different functional subtypes of fibroblasts have been suggested ranging from tumor-promoting to tumor-suppressing depending on the context [13, 24]. Novel promising technologies such as the mass cytometry CyTOF allow combining a large number of markers and allow isolating different phenotypic fibroblast subsets in order to perform functional experiments.

## Conclusions

Considering that the different CAF markers tested stained different cells, this suggests that a single fibroblast marker cannot recapitulate the heterogeneous composition of CAFs in the tumor stroma. Gene expression data showed an increased expression of CAF markers in stroma-high compared to stroma-low tumors. Histologically, the expression of FAP^+^ fibroblasts and the number of FSP1^+^/CD45^+^ cells were dependent on the spatial distribution. Stroma-high tumors showed a decreased number of FSP1^+^/CD45^+^ cells and an increased expression of FAP compared to stroma-low tumors. The prognostic relevance of FAP^+^ CAFs should be further explored as a stromal marker alone or to expand the TSR. Therapeutically targeting FAP^+^ CAFs may eventually promote anti-tumor growth in CRCs with high stromal content.

## Additional files

Additional file 1: **Figure S1.** Emission spectrum of the extracellular matrix-related and immune-related fibroblast markers used for spectral unmixing and representation of the single markers. **Figure S2.** Workflow of the measurement and analysis of the immunofluorescence staining. **Figure S3.** Western blot and immunohistochemical staining of fibroblast activated protein (FAP). **Table S1.** Patient characteristics of the LUMC cohort and stage III cohort. **Table S2.** Number and proportion of tumors expressing the different stromal markers in the tumor centre and at the invasive part following immunofluorescent quantification. (DOCX 4828 kb)

Additional file 2: Raw data and normalized data of the immunofluorescent quantification. (XLSX 70 kb)

## Abbreviations

CAF: Cancer-associated fibroblast
CRC: Colorectal cancer
ECM: Extracellular matrix
EMT: Epithelial-to-mesenchymal transition
FAP: Fibroblast associated protein
FFPE: Formalin fixed, paraffin-embedded
FSP1: Fibroblast specific protein 1, S100A4
H&E: Hematoxylin & eosin
LUMC: Leiden University Medical Center
PDGFRβ: Platelet-derived growth factor receptor
TSR: Tumor-stroma ratio
αSMA: α-smooth muscle actin
