# Supplementary material for: Increased expression of cancer-associated fibroblast markers at the invasive front and its association with tumor-stroma ratio in colorectal cancer
Source: BMC Cancer. 2019 Mar 29;19:284. doi: 10.1186/s12885-019-5462-2 (PMC6440123; doi:10.1186/s12885-019-5462-2)
Supplement: Supplementary file 1 — Figure S1. Emission spectrum of the extracellular matrix-related and immune-related fibroblast markers used for spectral unmixing and representation of the single markers. Figure S2. Workflow of the measurement and analysis of the immunofluorescence staining. Figure S3. Western blot and immunohistochemical staining of fibroblast activated protein (FAP). Table S1. Patient characteristics of the LUMC cohort and stage III cohort. Table S2. Number and proportion of tumors expressing the different stromal markers in the tumor centre and at the invasive part following immunofluorescent quantification. (DOCX 4828 kb) [file 12885_2019_5462_MOESM1_ESM.docx]

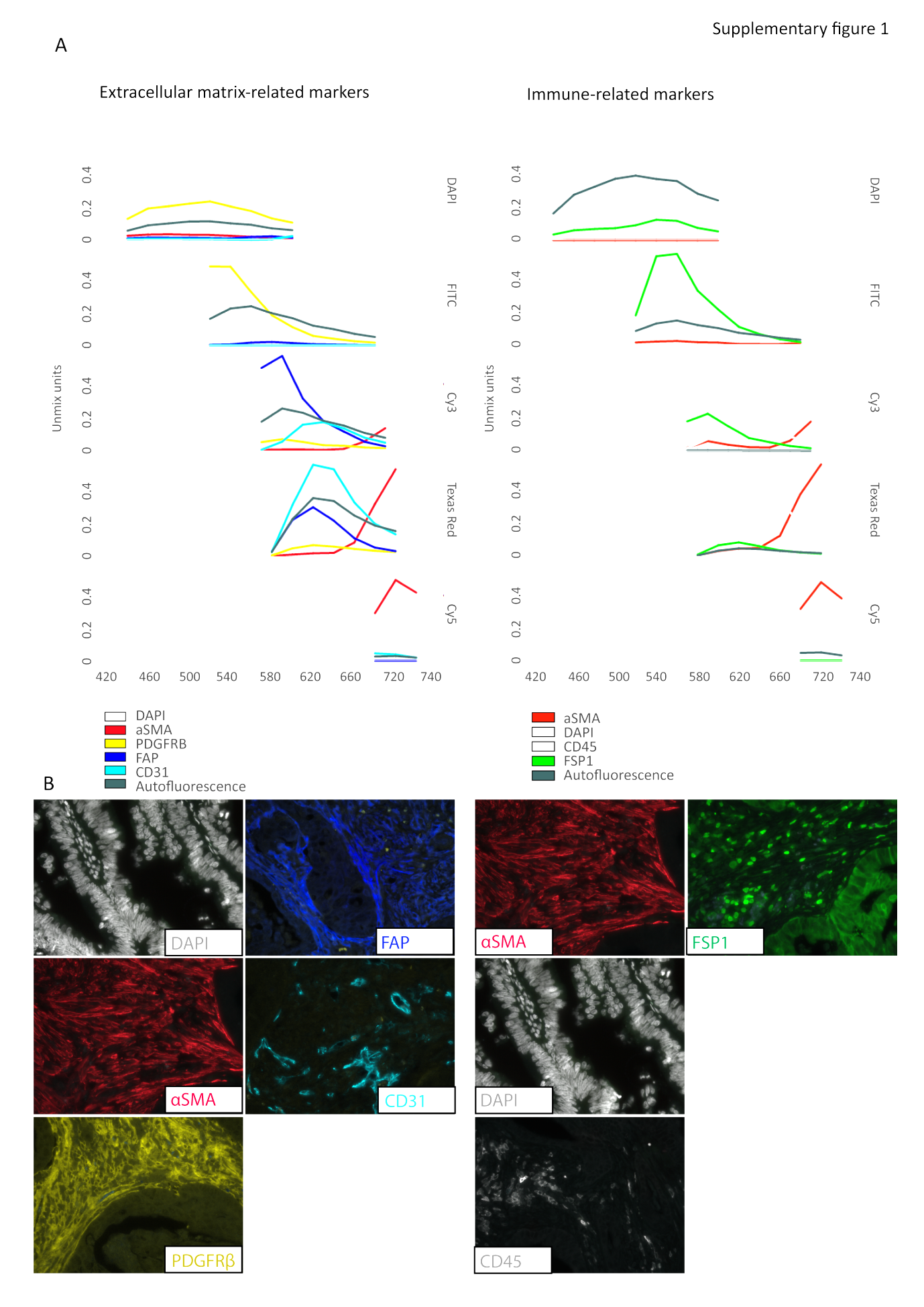


**Figure S1 – Emission spectrum of the extracellular matrix-related and immune-related fibroblast markers used for spectral unmixing and representation of the single markers.** The libraries with the emission spectrum of both panels of markers (A) were made using the signatures of single stained sections (B).


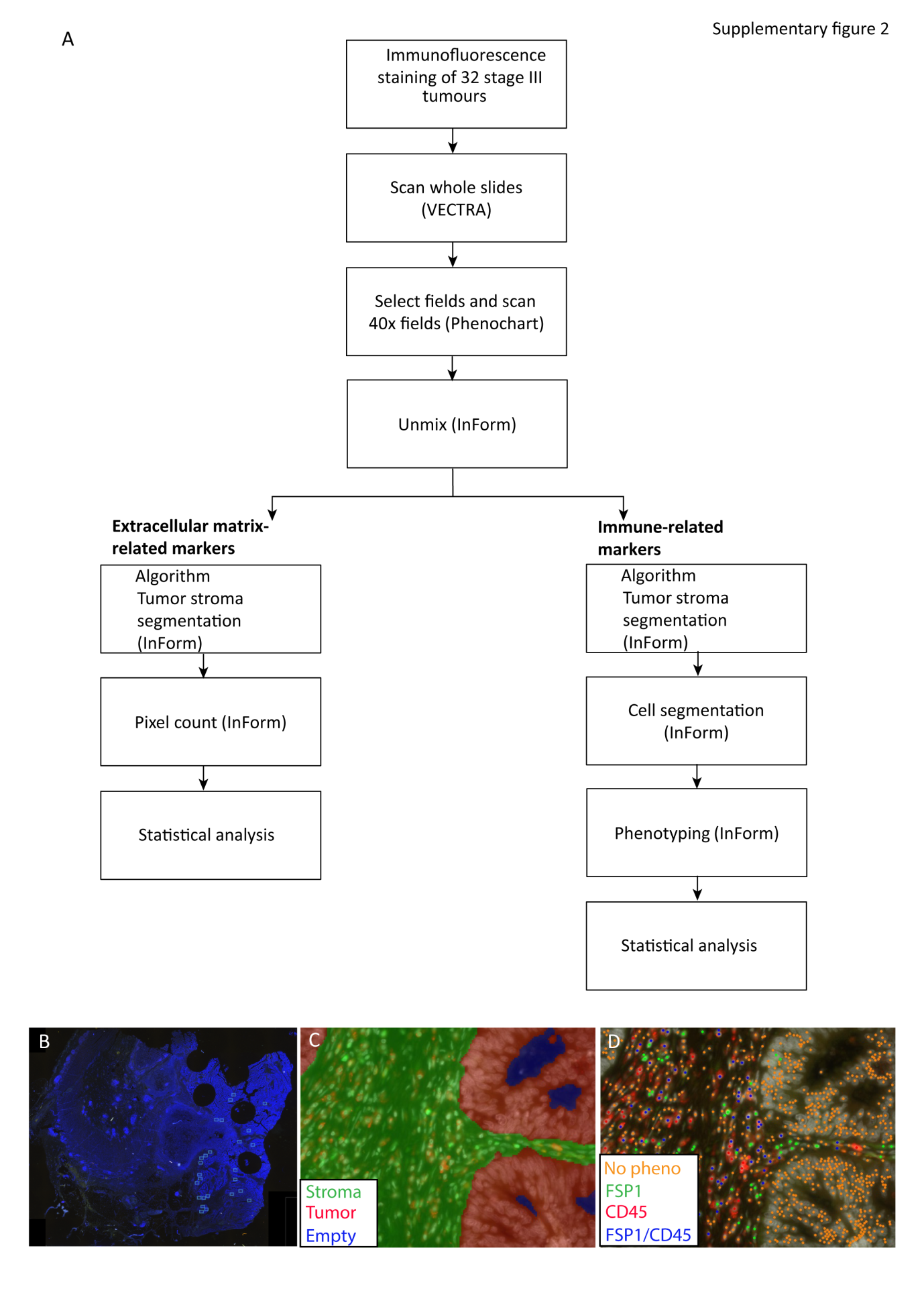


**Figure S2 – Workflow of the measurement and analysis of the immunofluorescence staining.** The workflow is depicted representing the staining of the sections, the scanning of the sections and the quantification of the spectrum according to both panels (A). 40x microscopical fields were selected on the unmixed scanned images (B). Following spectral unmixing, tissue segmentation algorithm identified regions of tumor (red), stroma (green) and empty (blue) (C).Within the stromal region, cells were recognized by their nuclei and phenotyped according to their marker (green = FSP1, red = CD45, blue = FSP1/CD45 and orange = no phenotype) (D).


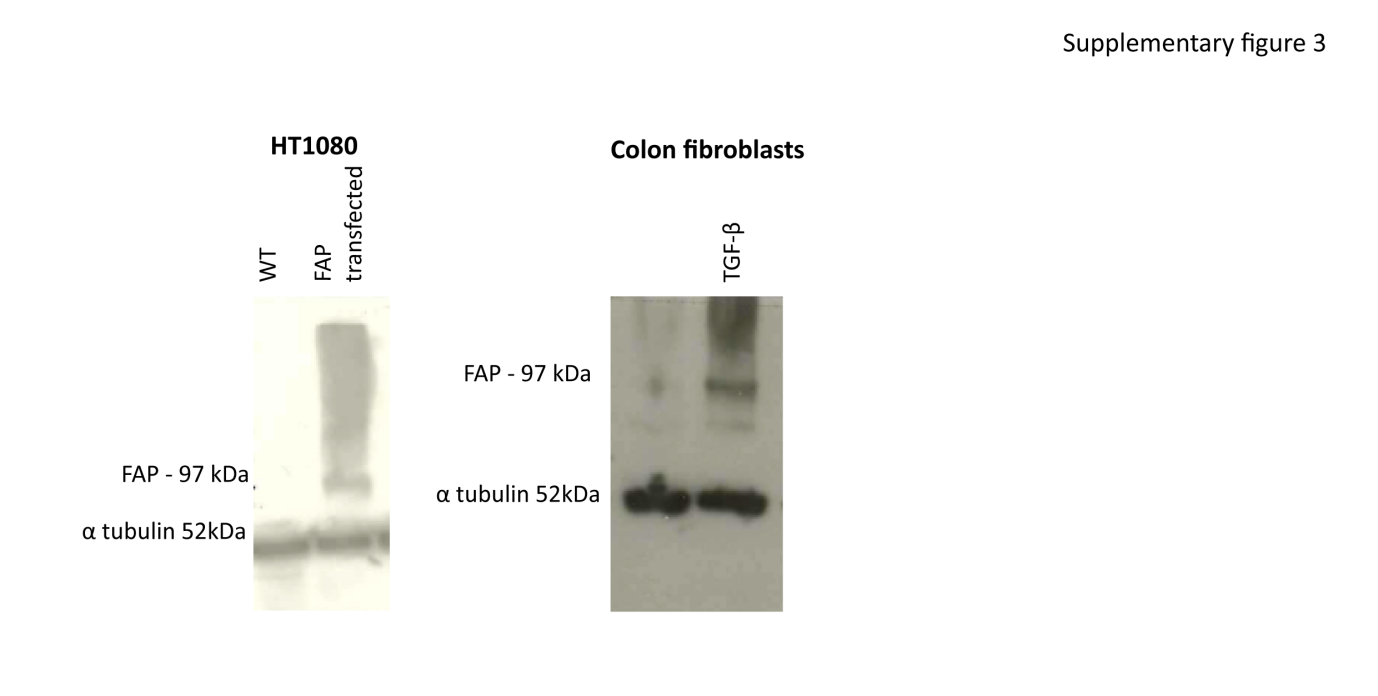


**B.**

**
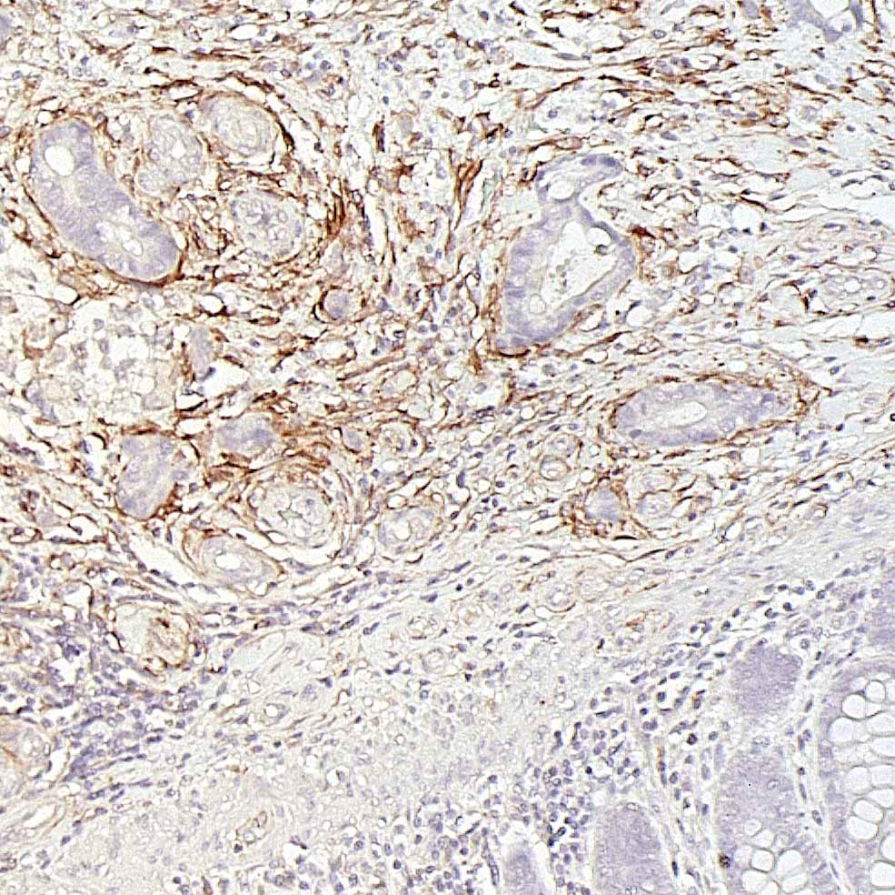
**

**Figure S3 – Western blot and immunohistochemical staining of fibroblast activated protein (FAP).** In (A), HT1080 wild type fibrosarcoma cells do not express any FAP protein at 97kDa while the FAP-transfected HT1080 shows high FAP protein. Human colon fibroblasts CCD-18Co shows low expression of FAP while TGFβ-stimulated fibroblasts shows strong FAP expression. Β-actin was used as a loading control. In (B), a stroma-high tumor shows the increased expression of FAP in CAFs surrounding tumor cells. However, there is no expression of FAP surrounding the epithelial cells of the crypts in the healthy tumor tissue (bottom right).

**Table S1. Patient characteristics**

1. **Patient characteristics of the LUMC cohort included in transcriptomic analysis**

|  |  | **Tumor-stroma ratio** | |  |
| --- | --- | --- | --- | --- |
|  |  | Stroma-low (%) N = 51 (71.8) | Stroma-high (%) N = 20 (28.2) | Total (%) N = 71 |
| **Gender** |  |  |  |  |
|  | Female | 26 (51) | 12 (60) | 38 (53.5) |
|  | Male | 25 (49) | 8 (40) | 33 (46.5) |
|  |  |  |  |  |
| **Mean age at surgery** | | 65.92 | 70.85 | 67.25 |
|  |  |  |  |  |
| **TNM stage** | |  |  |  |
|  | I | 8 (15.7) | 2 (10) | 10 (14.1) |
|  | II | 35 (68.6) | 4 (20) | 39 (54.9) |
|  | III | 8 (15.7) | 14 (70) | 22 (31) |
|  |  |  |  |  |
| **MSI status** | |  |  |  |
|  | MSS | 34 (68) | 14 (73.7) | 48 (67.6) |
|  | MSI-H | 16 (32) | 5 (26.3) | 21 (29.5) |
|  | Unknown | 1 | 1 | 2 (2.8) |
|  |  |  |  |  |
| **Location** |  |  |  |  |
|  | Colon | 40 (78.5) | 17 (85) | 57 (80.3) |
|  | Rectum | 11 (21.6) | 3 (15) | 14 (19.7) |
|  |  |  |  |  |
| **Vital status after 5 year** | |  |  |  |
|  | Alive | 40 (78.4) | 5 (25) | 45 (6.4) |
|  | Death | 11 (21.6) | 15 (75) | 26 (36.6) |
|  |  |  |  |  |
|  |  |  |  |  |

MSS, microsatellite stable; MSI-H, microsatellite instability - high

1. **Characteristics of patients with stage III colon cancer included in the cohort for immunofluorescence staining**

|  |  |  | **Tumor-stroma ratio** | | Total (%)  N = 32 |
| --- | --- | --- | --- | --- | --- |
|  |  |  | Stroma-low (%)  N = 18 (56.3) | Stroma-high (%)  N = 14 (43.7) |  |
|  | |  |  |  |  |
| **Gender** | |  |  |  |  |
|  | | Female | 8 (44.4) | 4 (28.6) | 12 (37.5) |
|  | | Male | 10 (55.6) | 10 (71.4) | 20 (62.5) |
|  | |  |  |  |  |
| **Mean age (years)** | | | 73.31 | 71.34 | 72.45 |
|  | |  |  |  |  |
| **TNM stage** | | |  |  |  |
|  | | IIIA | 3 (16.7) | 0 | 3 (9.4) |
|  | | IIIB | 8 (44.4) | 11 (78.6) | 19 (59.4) |
|  | | IIIC | 7 (38.9) | 3 (21.4) | 10 (31.3) |
| **MSI status** | | |  |  |  |
|  | | MSS | 9 (50.0) | 7 (50) | 16 (50.0) |
|  | | MSI-H | 1 (5.6) | 0 | 1 (3.1) |
|  | | Unknown | 8 (44.4) | 7 (50) | 15 (46.9) |
| **Location** | |  |  |  |  |
|  | | Colon | 18 | 11 (84.6) | 30 (93.8) |
|  | | Rectosigmoid | 0 | 2 (15.4) | 2 (6.2) |
|  | |  |  |  |  |
| **Morphology adenocarcinoma** | | |  |  |  |
|  | | Conventional | 13 (72.2) | 12 (85.7) | 25 (78.1) |
|  | | Cribriform | 2 (11.8) | 2 (14.3) | 4 (12.5) |
|  | | Mucinous | 1 (5.9) | 0 | 1 (3.1) |
|  | | Signet ring cell | 1 (5.9) | 0 | 1 (3.1) |
|  | | Intestinal-type | 1 (5.9) | 0 | 1 (3.1) |
|  | |  |  |  |  |
| **Differentiation grade** | | |  |  |  |
|  | | Grade 1 / well | 1 (5.6) | 1 (7.1) | 2 (6.3) |
|  | | Grade 2 / moderate | 9 (50.0) | 8 (57.1) | 17 (53.1) |
|  | | Grade 3 / poor | 4 (22.2) | 4 (28.6) | 8 (25.0) |
|  | | Unknown | 4 (22.2) | 1 (7.1) | 5 (15.6) |
|  | |  |  |  |  |
| **Adjuvant therapy** | | |  |  |  |
|  | | Yes | 10 (55.6) | 11 (78.6) | 21 (65.6) |
|  | | No | 8 (44.4) | 3 (21.4) | 11 (34.4) |
|  | |  |  |  |  |
| **Vital status after 5year** | | |  |  |  |
|  | | Alive | 14 (77.8) | 8 (57.1) | 22 (68.8) |
|  | | Death | 4 (22.2) | 6 (42.9) | 10 (31.2) |
|  | |  |  |  |  |
|  | | |  |  |  |

MSS, microsatellite stable; MSI-H, microsatellite instability - high

**Table S2 - Number and proportion (%) of tumors expressing the different stromal markers in the tumor center and at the invasive part following immunofluorescent quantification**

|  |  | **Tumor center** N (%) | **Invasive part** N (%) |
| --- | --- | --- | --- |
|  |  |  |  |
| **Extracellular matrix-related markers** | | N total = 27 | N total = 25 |
|  | aSMA | 27 (100) | 25 (100) |
|  | PDGFRb | 27 (100) | 25 (100) |
|  | FAP | 24 (88.9) | 25 (100) |
|  | CD31 | 26 (92.9) | 25 (100) |
|  |  |  |  |
| **Immune-related markers** | | N total = 25 | N total = 26 |
|  | CD45 | 25 (100) | 26 (100) |
|  | FSP1 | 19 (76) | 23 (88.5) |
|  | CD45/FSP1 | 24 (96) | 26 (100) |
|  |  |  |  |
